# Supplementary material for: A systematic review of (semi-)automatic quality control of T1-weighted MRI scans
Source: Neuroradiology. 2023 Dec 4;66(1):31–42. doi: 10.1007/s00234-023-03256-0 (PMC10761394; doi:10.1007/s00234-023-03256-0)
Supplement: Supplementary file 1 — Supplementary file1 (DOCX 22.3 KB) [file 234_2023_3256_MOESM1_ESM.docx]

# Supplementary materials

Table S.1.: Search strategy in PubMed

| **Search** | **Query** | **Results** |
| --- | --- | --- |
| #7 | #5 NOT #6 | 268 |
| #6 | "Review"[Publication Type] OR "Systematic Review" [Publication Type] OR "Meta-Analysis"[Publication Type] OR "Meta-Analysis as Topic"[Mesh] OR "meta-analysis"[tiab] OR "systematic review*"[tiab] OR "systematic literature review*"[tiab] OR "Letter"[Publication Type] OR "Editorial"[Publication Type] OR "Comment"[Publication Type] | 5,402,824 |
| #5 | #1 AND #2 AND #3 AND #4 | 326 |
| #4 | "Neurosciences"[Mesh] OR "Brain"[Mesh] OR "brain"[tiab] OR "head"[tiab] OR "cereb*"[tiab] OR Neuro*[tiab] OR "grey matter"[tiab] OR "gray matter"[tiab] | 3,713,585 |
| #3 | "Magnetic Resonance Imaging"[Mesh] OR "magnetic resonance imag*"[tiab] OR "MRI"[tiab] OR "MR imag*"[tiab] OR "NMR"[tiab] OR "nuclear magnetic resonance"[tiab] OR Neuroimag*[tiab] OR "Neuro imag*"[tiab] | 953,913 |
| #2 | "Quality Control"[Mesh] OR "quality control*"[tiab] OR "quality assessment*"[tiab] OR "quality check*"[tiab] OR "quality evaluat*"[tiab] | 127,939 |
| #1 | "Automation"[Mesh] OR Automat*[tiab] OR Toolbox*[tiab] OR Software*[tiab] OR Program*[tiab] OR "Artificial Intelligence"[Mesh] OR "Bayes Theorem"[Mesh] OR "Markov Chains"[Mesh] OR "Latent Class Analysis"[Mesh] OR AdaBoost[tiab] OR AI[tiab] OR Artificial-Intelligence*[tiab] OR autoencoder*[tiab] OR auto-encoder*[tiab] OR Back-propagation*[tiab] OR Bayesian-learning[tiab] OR gradient-boosting[tiab] OR CART[tiab] OR classification-algorithm*[tiab] OR Computational-Intelligen*[tiab] OR Computer-heuristic*[tiab] OR Computer-reasoning*[tiab] OR Computer-vision*[tiab] OR Connectionist-model*[tiab] OR decision-stump*[tiab] OR deep-belief-network*[tiab] OR Deep-learning[tiab] OR dirichlet[tiab] OR elastic-net[tiab] OR Ensemble[tiab] OR Expert-system*[tiab] OR Fuzzy-system[tiab] OR gaussianprocess*[tiab] OR generalized-additive-model*[tiab] OR generative-adversarial-network*[tiab] OR genetic-algorithm*[tiab] OR gradient-response-unit*[tiab] OR GRU[tiab] OR Heuristic-learning[tiab] OR Hierarchical-learning*[tiab] OR hierarchical-temporal-memor*[tiab] OR Image-Interpretation*[tiab] OR Image-recognition[tiab] OR Kernel-method*[tiab] OR LARS[tiab] OR LASSO[tiab] OR latent-class*[tiab] OR latent-process*[tiab] OR latent-variable*[tiab] OR LDA[tiab] OR Learning-algorithm*[tiab] OR learning-automat*[tiab] OR learning-machine*[tiab] OR learning-vector-quanti*[tiab] OR Least-Absolute-Shrinkage-and-Selection-Operator[tiab] OR least-angle-regression*[tiab] OR Logitboost[tiab] OR Long-short-term-memory[tiab] OR LSTM[tiab] OR Machine-intelligen*[tiab] OR Machine-learning[tiab] OR Machine-vision*[tiab] OR Markov-model*[tiab] OR Naïve-Bayes[tiab] OR Neural-network*[tiab] OR Deep-Boltzmann-Machine*[tiab] OR partial-least-squares-regression*[tiab] OR penalized-regression*[tiab] OR perceptron[tiab] OR PLSregression*[tiab] OR QDA[tiab] OR Qlearning[tiab] OR Q-learning[tiab] OR quadratic-classifier*[tiab] OR quadratic-discriminant*[tiab] OR random-forest*[tiab] OR reinforcement-learning[tiab] OR ridge-regression*[tiab] OR Rule-based[tiab] OR self-organising-map*[tiab] OR Speech-recognition[tiab] OR stacked-generali*[tiab] OR Supervised-learning[tiab] OR Support-vector-machine*[tiab] OR Support-vector-machine*[tiab] OR temporal-difference-learning[tiab] OR Textmining[tiab] OR Text-mining[tiab] OR Transfer-learning[tiab] OR Unsupervised-learning[tiab] OR XAI[tiab] OR "Machine Learning"[Mesh] OR "Machine Learning"[tiab] OR "machine intelligen*"[tiab] OR "machine vision*"[tiab] OR "machine learning"[tiab] OR "transfer learning"[tiab] OR "deep learning"[tiab] OR "neural network*"[tiab] OR "support vector machine*"[tiab] OR "Long short term memory"[tiab] OR "LSTM"[tiab] OR "supervised learning"[tiab] OR "unsupervised learning"[tiab] OR "reinforcement learning*"[tiab] OR "hierarchical learning*"[tiab] OR "Prediction model*"[tiab] OR "perceptron"[tiab] | 1,945,046 |

Table S.2.: Search strategy in Embase.com

| **Search** | **Query** | **Results** |
| --- | --- | --- |
| #6 | #5 AND ('article' / it OR 'article in press' / it OR 'conference paper' / it) | 496 |
| #5 | #1 AND #2 AND #3 AND #4 | 917 |
| #4 | 'neuroscience' / exp OR 'brain' / exp OR ('brain' OR 'head' OR 'cereb*' OR 'neuro*' OR 'grey matter' OR 'gray matter'):ti,ab,kw | 4,847,707 |
| #3 | 'nuclear magnetic resonance imaging' / exp OR ('magnetic resonance imag*' OR 'mri' OR 'mr imag*' OR 'nmr' OR 'nuclear magnetic resonance' OR 'neuroimag*' OR 'neuro imag*'):ti,ab,kw | 1,531,137 |
| #2 | 'quality control' / de OR (‘quality control*’ OR ‘quality assessment*’ OR ‘quality check*’ OR ‘quality evaluat*’):ti,ab,kw | 285,715 |
| #1 | 'automation' / exp OR 'automation':ti,ab,kw OR automat*:ti,ab,kw OR toolbox*:ti,ab,kw OR software*:ti,ab,kw OR program*:ti,ab,kw OR 'artificial intelligence' / exp OR 'artificial intelligence':ti,ab,kw OR 'bayes theorem' / exp OR 'bayes theorem':ti,ab,kw OR 'Markov chain' / exp OR 'markov chain*':ti,ab,kw OR 'latent structure analysis' / exp OR 'latent class analysis':ti,ab,kw OR adaboost:ti,ab,kw OR ai:ti,ab,kw OR 'artificial intelligence*':ti,ab,kw OR autoencoder*:ti,ab,kw OR 'auto encoder*':ti,ab,kw OR 'back propagation*':ti,ab,kw OR 'bayesian learning':ti,ab,kw OR 'gradient boosting':ti,ab,kw OR cart:ti,ab,kw OR 'classification algorithm*':ti,ab,kw OR 'computational intelligen*':ti,ab,kw OR 'computer heuristic*':ti,ab,kw OR 'computer reasoning*':ti,ab,kw OR 'computer vision*':ti,ab,kw OR 'connectionist model*':ti,ab,kw OR 'decision stump*':ti,ab,kw OR 'deep belief network*':ti,ab,kw OR dirichlet:ti,ab,kw OR 'elastic net':ti,ab,kw OR ensemble:ti,ab,kw OR 'expert system*':ti,ab,kw OR 'fuzzy system':ti,ab,kw OR gaussianprocess*:ti,ab,kw OR 'generalized additive model*':ti,ab,kw OR 'generative adversarial network*':ti,ab,kw OR 'genetic algorithm*':ti,ab,kw OR 'gradient response unit*':ti,ab,kw OR gru:ti,ab,kw OR 'heuristic learning':ti,ab,kw OR 'hierarchical temporal memor*':ti,ab,kw OR 'image interpretation*':ti,ab,kw OR 'image recognition':ti,ab,kw OR 'kernel method*':ti,ab,kw OR lars:ti,ab,kw OR lasso:ti,ab,kw OR 'latent class*':ti,ab,kw OR 'latent process*':ti,ab,kw OR 'latent variable*':ti,ab,kw OR lda:ti,ab,kw OR 'learning algorithm*':ti,ab,kw OR 'learning automat*':ti,ab,kw OR 'learning machine*':ti,ab,kw OR 'learning vector quanti*':ti,ab,kw OR 'least absolute shrinkage and selection operator':ti,ab,kw OR 'least angle regression*':ti,ab,kw OR logitboost:ti,ab,kw OR lstm:ti,ab,kw OR 'markov model*':ti,ab,kw OR 'naïve bayes':ti,ab,kw OR 'deep boltzmann machine*':ti,ab,kw OR 'partial least squares regression*':ti,ab,kw OR 'penalized regression*':ti,ab,kw OR perceptron:ti,ab,kw OR plsregression*:ti,ab,kw OR qda:ti,ab,kw OR qlearning:ti,ab,kw OR 'q learning':ti,ab,kw OR 'quadratic classifier*':ti,ab,kw OR 'quadratic discriminant*':ti,ab,kw OR 'random forest*':ti,ab,kw OR 'reinforcement learning':ti,ab,kw OR 'ridge regression*':ti,ab,kw OR 'rule based':ti,ab,kw OR 'self organising map*':ti,ab,kw OR 'speech recognition':ti,ab,kw OR 'stacked generali*':ti,ab,kw OR 'temporal difference learning':ti,ab,kw OR textmining:ti,ab,kw OR 'text mining':ti,ab,kw OR xai:ti,ab,kw OR 'machine learning' / exp OR 'machine learning':ti,ab,kw OR 'machine intelligen*':ti,ab,kw OR 'machine vision*':ti,ab,kw OR 'machine learning':ti,ab,kw OR 'transfer learning':ti,ab,kw OR 'deep learning':ti,ab,kw OR 'neural network*':ti,ab,kw OR 'support vector machine*':ti,ab,kw OR 'long short term memory':ti,ab,kw OR 'lstm':ti,ab,kw OR 'supervised learning':ti,ab,kw OR 'unsupervised learning':ti,ab,kw OR 'reinforcement learning*':ti,ab,kw OR 'hierarchical learning*':ti,ab,kw OR 'prediction model*':ti,ab,kw OR 'perceptron':ti,ab,kw | 2,706,198 |

Table S.3.: Search strategy in Web of Science

| **Search** | **Query** | **Results** |
| --- | --- | --- |
| #6 | #4 AND #3 AND #2 AND #1 and Article or Early Access or Data Paper (Document Types) | 252 |
| #5 | #1 AND #2 AND #3 AND #4 | 298 |
| #4 | TS=("brain" OR "head" OR "cereb*" OR Neuro* OR "grey matter" OR "gray matter") | 4,012,639 |
| #3 | TS=("magnetic resonance imag*" OR "MRI" OR "MR imag*" OR "NMR" OR "nuclear magnetic resonance" OR Neuroimag* OR "Neuro imag*") | 1,208,383 |
| #2 | TS=( "quality control*" OR "quality assessment*" OR "quality check*" OR "quality evaluat*") | 161,548 |
| #1 | TS=(Automat* OR Toolbox* OR Software* OR Program* OR AdaBoost OR AI OR Artificial-Intelligence* OR autoencoder* OR auto-encoder* OR Back-propagation* OR Bayesian-learning OR gradient-boosting OR CART OR classification-algorithm* OR Computational-Intelligen* OR Computer-heuristic* OR Computer-reasoning* OR Computer-vision* OR Connectionist-model* OR decision-stump* OR deep-belief-network* OR Deep-learning OR dirichlet OR elastic-net OR Ensemble OR Expert-system* OR Fuzzy-system OR gaussianprocess* OR generalized-additive-model* OR generative-adversarial-network* OR genetic-algorithm* OR gradient-response-unit* OR GRU OR Heuristic-learning OR Hierarchical-learning* OR hierarchical-temporal-memor* OR Image-Interpretation* OR Image-recognition OR Kernel-method* OR LARS OR LASSO OR latent-class* OR latent-process* OR latent-variable* OR LDA OR Learning-algorithm* OR learning-automat* OR learning-machine* OR learning-vector-quanti* OR Least-Absolute-Shrinkage-and-Selection-Operator OR least-angle-regression* OR Logitboost OR Long-short-term-memory OR LSTM OR Machine-intelligen* OR Machine-learning OR Machine-vision* OR Markov-model* OR Naïve-Bayes OR Neural-network* OR Deep-Boltzmann-Machine* OR partial-least-squares-regression* OR penalized-regression* OR perceptron OR PLSregression* OR QDA OR Qlearning OR Q-learning OR quadratic-classifier* OR quadratic-discriminant* OR random-forest* OR reinforcement-learning OR ridge-regression* OR Rule-based OR self-organising-map* OR Speech-recognition OR stacked-generali* OR Supervised-learning OR Support-vector-machine* OR Support-vector-machine* OR temporal-difference-learning OR Textmining OR Text-mining OR Transfer-learning OR Unsupervised-learning OR XAI OR "Machine Learning" OR "machine intelligen*" OR "machine vision*" OR "machine learning" OR "transfer learning" OR "deep learning" OR "neural network*" OR "support vector machine*" OR "Long short term memory" OR "LSTM" OR "supervised learning" OR "unsupervised learning" OR "reinforcement learning*" OR "hierarchical learning*" OR "Prediction model*" OR "perceptron") | 4,161,582 |

Table S.4: T1w MRI sequences

| **Article** | **Dataset** | | | |
| --- | --- | --- | --- | --- |
|  | **Name** | **Acquisition** | **Sequence** | **Field Strength** |
| [20] | NA | 3D | mix* | 3T |
|  | NA | 3D | mix* | 3T |
|  | NA | 3D | mix* | 3T |
| [21] | NA | 2D | FSE | 3T |
| [22] | ADNI | 3D | Inversion recovery spoiled gradient | 3T |
|  | ABIDE | 3D | Inversion recovery spoiled gradient | 3T |
| [24] | TRACK-TBI | 3D | Inversion recovery spoiled gradient | 3T |
| [26] | ADNI | 3D | Inversion recovery spoiled gradient | 3T |
|  | ABIDE | 3D | Inversion recovery spoiled gradient | 3T |
| [28] | ADNI | 3D | Inversion recovery spoiled gradient | 3T |
|  | NeuroRX | 3D | Inversion recovery spoiled gradient | 3T |
| [29] | ADNI | 3D | Inversion recovery spoiled gradient | 3T |
|  | NeuroRX | 3D | Inversion recovery spoiled gradient | 3T |
| [27] | NeuroRX | 3D | Inversion recovery spoiled gradient | 3T |
| [32] | Generation R Wave I | 3D | Inversion recovery spoiled gradient | 3T |
|  | Generation R Wave II | 3D | Inversion recovery spoiled gradient | 3T |
|  | NHGRI | 3D | Inversion recovery spoiled gradient | 3T |
|  | GUSTO | 3D | Inversion recovery spoiled gradient | 3T |
| [33] | MNI | 2D | not reported | not reported |
| [11] | UK Biobank | 3D | Inversion recovery spoiled gradient | 3T |
| [12] | ABIDE | 3D | Inversion recovery spoiled gradient | 3T |
|  | DS030 | 3D | Inversion recovery spoiled gradient | 3T |
| [30] | CBDB Sibling Study | 3D | SPGR | 3T |
| [19] | NA | 3D | Inversion recovery spoiled gradient | 3T + 1.5T |
| [8] | NA | 3D | Inversion recovery spoiled gradient | 3T |
|  | NA | 3D | Inversion recovery spoiled gradient | 3T |
|  | NA | 3D | Inversion recovery spoiled gradient | 3T |
|  | NA | 3D | Inversion recovery spoiled gradient | 3T |
| [23] | HBN | 3D | Inversion recovery spoiled gradient | 3T |
| [25] | NA | 2D | FSE | 3T |
| [31] | ABIDE | 3D | Inversion recovery spoiled gradient | 3T |

Inversion recovery spoiled gradient-like sequences include MPRAGE and IR-SPGR

FSE = Fast Spin Echo, MPRAGE = Magnetization Prepared Rapid Gradient Echo, SPGR = Spoiled Gradient echo

* a mix of sequences has been used, but it was not specified which ones exactly
